# Co-migration fidelity at a stopover site increases over time in African–European migratory landbirds

**DOI:** 10.1098/rsos.221043

**Published:** 2023-08-30

**Authors:** Bruno Bellisario, Massimiliano Cardinale, Ivan Maggini, Leonida Fusani, Claudio Carere

**Affiliations:** ^1^ Department of Ecological and Biological Sciences, University of Tuscia, Viterbo, Italy; ^2^ Swedish University of Agricultural Sciences, Department of Aquatic Resources, Institute of Marine Research, Lysekil, Sweden; ^3^ Konrad-Lorenz Institute of Ethology, University of Veterinary Medicine, Vienna, Austria; ^4^ Department of Behavioural and Cognitive Biology, University of Vienna, Vienna, Austria

**Keywords:** stopover, co-occurrence networks, migration phenology, avian assemblages, interspecific interactions

## Abstract

Migratory species are changing their timing of departure from wintering areas and arrival to breeding sites (i.e. migration phenology) in response to climate change to exploit maximum food availability at higher latitudes and improve their fitness. Despite the impact of changing migration phenology at population and community level, the extent to which individual and species-specific response affects associations among co-migrating species has been seldom explored. By applying temporal co-occurrence network models on 15 years of standardized bird ringing data at a spring stopover site, we show that African–European migratory landbirds tend to migrate in well-defined groups of species with high temporal overlap. Such ‘co-migration fidelity’ significantly increased over the years and was higher in long-distance (trans-Saharan) than in short-distance (North African) migrants. Our findings suggest non-random patterns of associations in co-migrating species, possibly related to the existence of regulatory mechanisms associated with changing climate conditions and different uses of stopover sites, ultimately influencing the global economy of migration of landbirds in the Palearctic–African migration system.

## 1. Introduction

Migration represents a key life-history event for thousands of species and is typically associated with the temporal and geographic variation in resource availability [1]. Alongside endogenous (e.g. circannual rhythms) and exogenous (e.g. photoperiodic cues) mechanisms [2,3], favourable environmental conditions and food availability *en route* are key elements driving the timing of migration in birds [4–7]. Climate change may pose serious problems to migrants spending different parts of their annual cycle in different parts of the world as it drives the restructuring of important phenological events [8–10]. Overall, differences in the extent of climatic change along migratory routes, as well as at wintering, stopover and breeding areas [11–13], increase the chance of phenological mismatch (i.e. when interacting species change the timing of regularly repeated life cycle phases at different rates), thus influencing the interactions among co-occurring species including their food sources, predators, and competitors [14,15]. Indeed, migrations involve the simultaneous movement of multiple species at once connecting separated and diverse communities whose migratory routes often converge in space and time [16], resulting in direct and indirect ecological interactions such as predation, social interactions and competition for resources [17–20].

Within-species variability in migratory behaviour can influence the degree of association between individuals of a population in different seasons (i.e. the strength of migratory connectivity), as well as possible interactions with resident and other migratory species. Advances in the date of departure from wintering areas in response to endogenous and/or exogenous factors reflecting changing climate conditions have been observed in several species [21–23]. Populations of origin, sex, age and ecological requirements of individuals might all affect the likelihood of species to change migration phenology [24–29], leading in some cases to differential (asynchronous) migration patterns where subgroups of a population migrate at different times [30]. This could have profound effects on community processes and ecosystem functions as, for instance, nutrient cycling and primary productivity [31]. However, despite the ecological importance of interactions between co-migrants, we still lack a proper understanding of how long-term phenological changes affect the restructuring of avian assemblages *en route*.

The change in spring passage of African–European migratory landbirds is a well-documented phenomenon, with the peak passage in the Mediterranean basin advancing by up to one day per year across the last two decades [23]. Cues available to adjust departure vary at different rates depending on wintering location and habitat, and adjustments to changing climate might be less pronounced in species wintering the furthest away from their breeding grounds (e.g. the Sahel and the tropics) [23]. Although the causes and consequences of *en route* adjustments to migration are well known at the level of single species and/or populations, a full-community perspective involving the simultaneous movement of migrants has been seldom explored. Incorporating the complexity of migrant–migrant relationships could uncover novel interactions or shared environmental responses among species and predict unexplored consequences of global change on migratory systems, ultimately improving conservation efforts.

Investigating the causes and the effects of changing migration phenology from a community point of view entails inherent difficulties associated with the need to constantly track and monitor animals across oceans and continents [16]. A further source of uncertainty derives from the need to appropriately model multi-species temporal movements to identify recurrent patterns in the aggregation of species and forecast the consequences of climate change on migrations. Within this context, network analysis can be a valuable tool to elucidate mechanisms driving the temporal assemblage of species [32]. Time series data can be used to reconstruct temporal co-occurrence networks, reflecting the likelihood of correlation among nodes (i.e. species) able to reveal successions of temporal patterns (e.g. migratory activity), corresponding to changes in behavioural modes (e.g. migration phenology) that can be attributed to specific spatio-temporal events (e.g. climate change). Temporal co-occurrence networks can thus help illustrate complex properties of animal movements with simple graphical metrics [32], although the actual ecological meaning of revealed links must be critically evaluated (see [33] and references therein).

In this study, we propose a novel method using time series data of standardized bird ringing activity and network theory to understand the effects of changing migration phenology on the co-occurrence of African–European migratory landbirds at a Mediterranean spring stopover island. Data were used to construct temporal co-occurrence networks and derive a measure of ‘co-migration fidelity’, to test for the existence of well-defined groups of species always migrating together. We discuss the possible causes of co-migration by considering the wintering grounds as a proxy of migration strategy (i.e. long- and short-distance migrants), and the possible consequences for coexistence by considering the feeding strategies of birds in relation to the multiple functions a stopover area can supply. We hypothesize that co-migration fidelity should be influenced by the responses of birds to the effect of changing environmental conditions at wintering grounds, by altering their migration phenology in relation to their migration strategy (i.e. North African versus trans-Saharan species). Moreover, considering that refuelling is not the primary reason to stop over at our study site, and that only a very small fraction (usually below 2%) stays for longer than one day [34], co-existence at stopover should not be regulated by mechanisms aimed at minimizing competition and, therefore, we should observe little or no differences in the co-occurrence of species with different foraging strategies. We argue that our approach can promote the development of comparative studies in other migratory systems and bring new insights into the causes and effects of global change on migratory activity.

## 2. Material and methods

### 2.1. Sampling site and bird ringing activity

**Figure 1.**
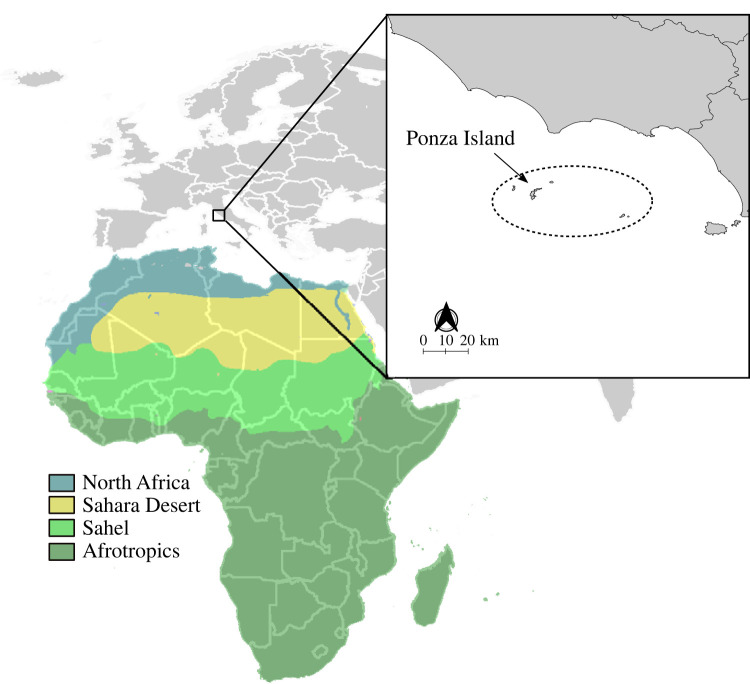
Geographical framework of the study area within the Palearctic–African migration system. The dotted circle in the box shows the Pontine islands system with the exact location of Ponza. Colours in the map show a synthetic view of the main African ecoregions corresponding to the supposed wintering areas of species analysed in this study.

The study was conducted on Ponza (figure 1), a small island in the Tyrrhenian Sea with an extent of *ca* 9.87 km^2^, located about 50 km off the western coast of Italy (40°55′ N, 12°58′ E), where we have been monitoring spring migration through capture and ringing since 2002 (http://www.inanellamentoponza.it). Ponza is located along one of the main Mediterranean migratory routes and attracts a large number of African–European migratory landbirds during spring migration [23]. The bird catching season for birds wintering in Africa and using the small islands system of the central Mediterranean Sea as stopover areas during their spring migration (figure 1) extends from the beginning of March to the end of May [35]. Birds were captured using mist nets operated every day except for days with heavy rain or strong winds (greater than 15 knots). The number of days with reduced effort was less than 1% of the total. An average length of 227 m of mist nets were checked hourly from dawn until one hour after dusk throughout the study period.

We kept the net brand and model (Lavorazione Reti Bonardi, Monte Isola, BS, Italy, http://www.vbonardi.it/; 2.4 m height, 16 mm mesh size), as well as the vegetation height at the catching site constant throughout the entire study period. After being captured, birds were ringed and measured using standard EURING procedures [36]. Here we used the daily abundances of the 31 most abundant non-resident species landing on Ponza (table 1) over a period of 15 years (2007 to 2021), during which the procedures were fully standardized.

**Table 1.** List of the 31 most abundant species ringed at the stopover site of Ponza Island during the period 2007–2021.

species	wintering area	foraging niche	number of sampled individuals		
			total	mean (± s.d.) across years	range [min–max]
*Acrocephalus arundinaceus* (great reed warbler)	Tropical	invertivore glean arboreal	1156	25.2 ± 11.391	[13–58]
*Acrocephalus schoenobaenus* (sedge warbler)	Sahel	invertivore glean arboreal	378	77.067 ± 48.792	[40–236]
*Anthus trivialis* (tree pipit)	Tropical	invertivore ground	2197	146.467 ± 44.171	[72–207]
*Delichon urbica* (common house martin)	Tropical	invertivore sally air	1283	85.534 ± 49.393	[21–187]
*Erithacus rubecula* (European robin)	North Africa	invertivore ground	14 948	996.534 ± 877.128	[84–2599]
*Ficedula albicollis* (collared flycatcher)	Tropical	invertivore sally air	11 504	121.734 ± 99.349	[5–279]
*Ficedula hypoleuca* (European pied flycatcher)	Tropical	invertivore sally air	1826	766.934 ± 271.304	[369–1393]
*Fringilla coelebs* (common chaffinch)	North Africa	invertivore ground	421	28.067 ± 20.537	[1–58]
*Hippolais icterina* (icterine warbler)	Tropical	invertivore glean arboreal	18 850	1256.667 ± 706.695	[325–2922]
*Hirundo rustica* (barn swallow)	Sahel	invertivore sally air	6402	426.8 ± 132.102	[208–700]
*Jynx torquilla* (Eurasian wryneck)	Sahel	invertivore ground	501	33.4 ± 15.263	[17–67]
*Lanius senator* (woodchat shrike)	Tropical	invertivore ground	621	41.4 ± 14.603	[17–71]
*Luscinia megarhynchos* (common nightingale)	Tropical	invertivore ground	1976	131.733 ± 50.941	[56–214]
*Merops apiaster* (European bee-eater)	Sahel	invertivore sally air	757	50.466 ± 26.848	[21–114]
*Muscicapa striata* (spotted flycatcher)	Sahel	invertivore sally air	9500	633.333 ± 246.003	[187–1108]
*Oenanthe oenanthe* (northern wheatear)	Sahel	invertivore sally ground	2222	148.133 ± 59.677	[67–259]
*Oriolus oriolus* (Eurasian golden oriole)	Tropical	omnivorous arboreal	1343	89.533 ± 37.557	[39–182]
*Phoenicurus ochruros* (black redstart)	North Africa	invertivore sally ground	2750	183.333 ± 142.749	[6–397]
*Phoenicurus phoenicurus* (common redstart)	Sahel	invertivore sally ground	6728	448.533 ± 255.998	[155–1118]
*Phylloscopus collybita* (common chiffchaff)	North Africa	invertivore glean arboreal	5880	392 ± 288.111	[88–1050]
*Phylloscopus sibilatrix* (wood warbler)	Tropical	invertivore glean arboreal	15 402	1026.8 ± 285.567	[597–1430]
*Phylloscopus trochilus* (willow warbler)	Sahel	invertivore glean arboreal	13 466	897.733 ± 315.275	[424–1421]
*Saxicola rubetra* (Whinchat)	Tropical	Invertivore sally ground	13 392	892.8 ± 432.842	[7–1646]
*Saxicola torquata* (common stonechat)	North Africa	invertivore sally ground	780	52 ± 60.523	[2–184]
*Streptopelia turtur* (European turtle dove)	Sahel	granivore ground	724	48.266 ± 36.526	[18–139]
*Sylvia atricapilla* (Eurasian blackcap)	North Africa	invertivore glean arboreal	13 885	925.666 ± 1738.913	[29–5614]
*Sylvia borin* (garden warbler)	Tropical	invertivore glean arboreal	35 655	2377 ± 1485.051	[337–5967]
*Sylvia cantillans* (subalpine warbler)	Sahel	invertivore glean arboreal	14 117	941.133 ± 659.287	[259–2789]
*Sylvia communis* (common whitethroat)	Sahel	invertivore glean arboreal	25 358	1690.533 ± 1023.444	[40–3589]
*Turdus philomelos* (song thrush)	North Africa	invertivore ground	1985	132.333 ± 146.932	[16–477]
*Upupa epops* (Eurasian hoopoe)	Sahel	invertivore ground	526	35.066 ± 18.525	[15–76]

### 2.2. Network inference and modularity

For each year of survey (*n* = 15), we infer the co-occurrence network structure (i.e. the reconstruction of all possible relationships between nodes in a network) by calculating the time-lagged Spearman rank correlations using the daily abundances of each species with each other in the previous time step (i.e. the lag-1 correlation [37]). To avoid spurious correlations due to extremely low abundances, we removed from computation the species for which the total number of individuals in each year was below the 25th percentile of the entire distribution. We used the packages ‘Hmisc’ [38] and ‘qvalue’ [39] of R (version 4.3.0) [40] to adjust the significance of correlations using the Bonferroni–Holm procedure [41]. Links (i.e. co-occurrences) between species were considered significant for values of *r* > 0.5 and *p* < 0.05. We used unweighted links, where co-occurrences were considered irrespective of the strength of the correlation.

We used modularity (*Q*) to detect groups of species characterized by common migration timing. In our case, the higher the value of *Q*, the greater the tendency of networks to cluster into subgroups of species having high temporal co-occurrence. From an ecological point of view, modularity is a straightforward structural property of networks able to detect species with similar responses to the main spatial, environmental and biotic (e.g. species interactions) processes structuring biodiversity [42–44]. We used an eigenvector-based maximizing algorithm (i.e. the Louvain algorithm [45]) to measure *Q*, the degree to which a network subdivides in densely connected groups of nodes (aka modules) with only sparser connections between groups [46]. We considered networks with *Q* > 0.3 as having a strong modular subdivision [46].

The yearly measured modularity values were filtered by means of a simple moving average (SMA) to reduce background noise in the original time series and emphasize trends. We used SMA with an automated selection procedure based on the Akaike information criterion to find the optimal order of the moving average [47]. Modularity was measured using the function ‘cluster_louvain’ [45] in the package ‘igraph’ [48] of R, while we used the functions ‘sma’ and ‘sen.slope’ in the packages ‘smooth’ [47] and ‘trend’ [49], respectively, to smooth the time series and measure the Mann–Kendall statistics.

### 2.3. Co-migration fidelity

We defined ‘co-migration fidelity’ as the frequency with which two species tended to co-occur in the same module over the years, measured by using the normalized mutual information (*nmi*) [50]. Basically, *nmi* assesses whether two classifications (i.e. module affiliation in different years) on the same set (i.e. species) explain one another, providing a robust metric for comparing network partitions as it is not affected by the number and size of modules found in each network. The normalized mutual information is bounded between 0 and 1, so that if two species were always part of the same module over the years (i.e. perfect congruence, high fidelity and similar changes in migration timing), then *nmi* = 1; conversely, if two species never co-occurred within the same module over the years (i.e. independent, no fidelity and different changes in migration timing), then *nmi* = 0. Data were organized in a matrix whose elements represented the module affiliation in each year of sampling (columns) for each species (rows), measuring all *nmi* values between rows to obtain a symmetric similarity matrix expressing the pairwise mutual information among species.

We used the permutational multivariate analysis of variance using distance matrices for partitioning the measured normalized mutual information among sources of variation given by wintering areas and foraging niches. We divided the species in three main groups (table 1) corresponding to those wintering either north of the Sahara Desert (North African), in the sub-Saharan belt extending between the Sahara Desert and the Sudan savannah (Sahel), or in the Afrotropical ecozone (Tropical) [23]. Following Pigot *et al*. [51], species were also characterized by main foraging niches (table 1). The normalized mutual information and permutational multivariate analysis of variance were calculated using the function ‘compare’ and ‘adonis2′ in the ‘igraph’ and ‘vegan’ [48,52] packages of R, respectively.

## 3. Results

### 3.1. Bird ringing data

A total of 226 533 individuals were ringed over the 15 years of field activity, with an average (± s.d.) of 15 117 ± 4031 per year and a minimum of 8861 in 2010 and a maximum of 23 186 in 2015. Three main families accounted for 84% of the total: Sylviidae (four species, 40%), Muscicapidae (seven species, 29%) and Phylloscopidae (three species, 15%) (table 1).

### 3.2. Network modularity

**Figure 2.**
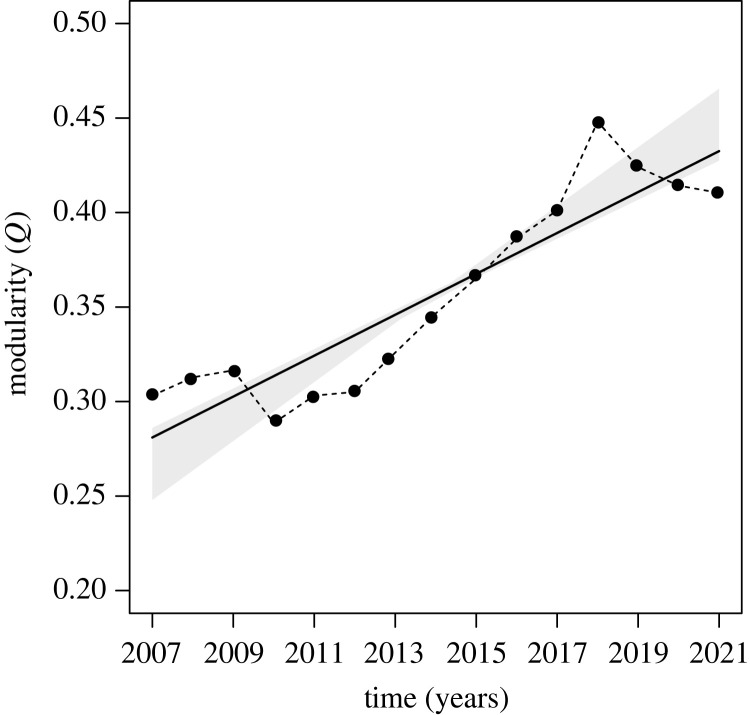
Trend of modularity (*Q*), showing the degree to which temporal co-occurrence networks subdivide in groups of species characterized by common migration timing. The higher the value, the greater the tendency of networks to cluster into subgroups of species having high temporal co-occurrence. The dashed black line indicates the original time series, while the solid line and the shaded grey area show the measured trends and uncertainty in trend slopes, respectively.

Overall, temporal co-occurrence networks always showed relatively strong modular structures, with an average *Q* (± s.d.) of 0.357 ± 0.053 and ranging from a minimum of 0.288 in 2010 and a maximum of 0.448 in 2018, while showing overall a significant increase over the years (Mann–Kendall trend test *z* = 2.969, *p* = 0.003) (figure 2).

### 3.3. Co-migration fidelity

**Figure 3.**
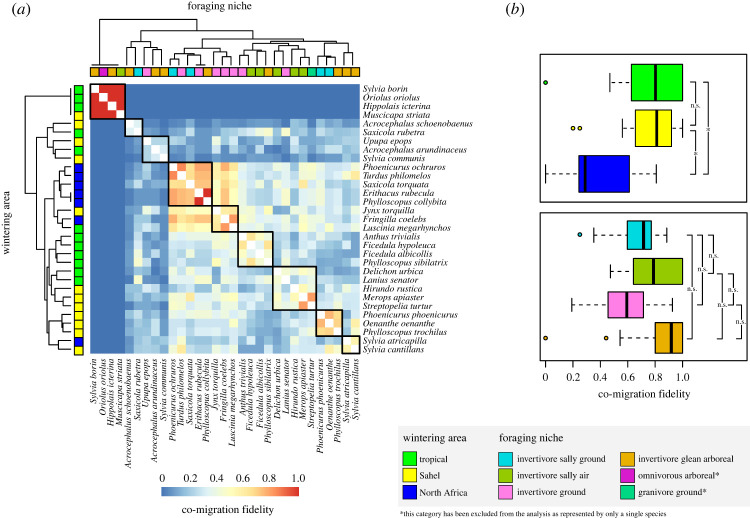
(*a*) Heatmap showing the species' co-migration fidelity (i.e. the frequency with which species co-occurred in the same module over the years), with colours corresponding to the measured normalized mutual information, *nmi* (see the legend at the bottom of the heatmap). Dendrograms above and alongside the heatmap represent the cluster subdivision of species based on the *nmi* values, using the farthest neighbour method to calculate distance between clusters in hierarchical clustering. Coloured boxes below and alongside dendrograms show to which foraging niche and wintering area, respectively, a species belongs (see the legend at the bottom of the heatmap). Thick-edged squares within the heatmap show the main cluster subdivision of species obtained by cutting the dendrogram at a pre-defined height of 0.5, corresponding to a minimum level of similarity of 50%. (*b*) Boxplot showing the *nmi* distribution in species having different wintering areas and foraging niches. Black lines alongside boxplots indicate the significance levels (ns, not significant; *p < 0.05). Two foraging niche categories (granivore ground and omnivorous arboreal) were not included in the analysis due to the presence of only one species each (*Streptopelia turtur* and *Oriolus oriolus*).

The pairwise co-migration fidelity, measured by the normalized mutual information (*nmi*), showed a well-defined pattern, with species subdividing in nine main clusters (figure 3*a*). Interestingly, one main cluster (figure 3*a*) was characterized by species having high values of the *nmi* index (*Sylvia borin*, *Hippolais icterina*, *Muscicapa striata*, *Oriolus oriolus*), indicating their tendency to always co-occur in the same module over the years, with little or no temporal overlap with all other species. Other species, such as the black redstart (*Phoenicurus ochruros*), the song thrush (*Turdus philomelos*), the common stonechat (*Saxicola torquata*), the European robin (*Erithacus rubecula*) and the common chiffchaff (*Phylloscopus collybita*), showed more variable *nmi* index, with partial temporal overlap with all other species (figure 3*a*). Co-migration fidelity significantly differed between species wintering in different areas, with birds wintering in North Africa showing on average lower values of *nmi* compared to those wintering in the Sahel (*F* = 2.793, *p* = 0.027) and Tropical ecozones (*F* = 2.793, *p* = 0.003; figure 3*b*). We did not find significant differences with respect to foraging niches (*F* < 1.917 and *p* > 0.108 for all pairwise comparisons; figure 3*b*).

## 4. Discussion

By applying network models to a long-term series of migratory passerines at a Mediterranean stopover site during spring, we showed that changes in the migration timing of African–European landbirds [23] lead to novel patterns in the assemblage of co-occurring migrants. Temporal co-occurrence networks always had high values of modularity, which arises from a general tendency of birds to cluster in groups of species having common migration timing. Documented differences in the rescheduling of migratory activity between short (i.e. North African) and long-distance migrants (i.e. trans-Saharan) [23] enhance this pattern, resulting in an increasing modularity and co-migration fidelity over the years in trans-Saharan compared to North African migrants. As a consequence, birds at the stopover area have a general tendency to create well-defined modules of species characterized by similar responses to changes in migration phenology.

Spring migration phenology of birds crossing the Mediterranean Sea can be related to specific ecological factors associated with travel distance and competition for nesting sites, as well as to the degree of sexual dimorphism [53,54]. For instance, species wintering closer to the breeding areas (e.g. North Africa) usually have faster adaptive responses due to their ability to better track changing conditions at the breeding grounds [55]. Latitudinal differences in wintering sites might thus determine the phenology of spring migration because species wintering north of Sahara are advancing their peak date of passage compared to those spending the non-breeding season in sub-Saharan Africa [23]. Moreover, recent findings have shown a substantial increase (up to one day per year) of the migration window for species wintering south of the Sahara Desert, possibly related to more favourable conditions at wintering areas that allow faster refuelling, earlier departure, and/or longer residence time [23]. The strong modular subdivision observed over the study period (with the only exception of 2010) suggests that passerine species moving within the Palaearctic–African flyway during spring migration always followed a well-defined order of migration, a tendency likely to be predominant in those species forced to cover larger distances to reach their breeding areas.

How migratory birds manage to detect and adapt to changing environmental conditions during their journey to breeding areas remains an open question related primarily to the relative importance of both micro-evolutionary change and phenotypic plasticity [56]. Phenotypic plasticity may provide faster adaptive responses, especially for species travelling shorter distances, and capable of immediate responses to changing environmental conditions [57]. As a result, short-distance migrants are less prone to demographic declines compared to long-distance migrants [15], a pattern already documented in many landbirds passing through Ponza [35]. This might have led to a change in the diversity of annual captures, with a trend towards the dominance of fewer species (mostly those departing from North Africa) while others become rarer (trans-Saharan migrants), possibly increasing network modularity over the years. Our current results do not elucidate this hypothesis, as species might have experienced the same annual trend but in different time windows during the catching season because of asynchrony in the medians of their daily passages, leading to uncorrelated patterns of association. Further investigations are needed for a better understanding of how demographic trends in populations influence modularity in co-occurrence networks.

### 4.1. Pattern of co-migration fidelity

The increasing trend in modularity was associated with a general tendency of birds to group together in modules composed largely of the same species over the years. This indicates that the passage and landing on the stopover area of Ponza are characterized by a strong co-migration fidelity, especially in long-distance migrants, a pattern likely associated with the emergence of common responses to different environmental conditions experienced by birds during their journey to the breeding grounds. Overall, behavioural decisions related to the starting of the spring migration are based on a complex set of cues to fatten and depart from the non-breeding area, often linked to exogenous and endogenous information such as weather conditions and individual-specific traits like age, sex, body size and fuel store [2,3].

Geographical mismatches in climate variability and weather conditions along the migratory route can affect the birds' migration phenology in different ways. For instance, the different environmental conditions experienced over recent years by African wintering grounds (e.g. the re-greening of the Sahel and the drying of North Africa) may result in regional differences in the phenological adjustment of birds using Ponza as stopover area [23]. It is possible that under less favourable conditions birds may be more sensitive to the negative effects of density-dependent mechanisms on foraging activity [58], limiting their residence time due to the competitive pressure triggered by a reduced availability of resources. Under this scenario, migrants might have a lower tendency to co-migrate with other species, being limited by the need to outcompete other species and, therefore, move quickly to breeding grounds. Moreover, in birds wintering in close proximity to their breeding area, local environmental cues such as temperature and precipitation could be more important than endogenous circannual rhythms, leading to higher intra- and interspecific variability in the timing of migration in short- versus long-distance migrants [59]. This would explain why, for instance, North African migrants showed lower co-migration fidelity than trans-Saharan ones, a pattern likely related to more flexible responses to the effect of changing climate conditions *en route*, possibly influencing the repeatability and synchrony of migration phenology over time.

### 4.2. Possible consequences of co-occurrence at stopover site

Although co-occurrence patterns cannot be used to infer actual interspecific interactions (see [33] and references therein), they can reveal potential for interferences among species, especially when forced to share small areas in limited time frames, such as at migratory bottlenecks [35]. This is the case of stopover areas, where birds stop to rest, refuel and seek shelter during their migration, and where the decision on where, when and how long to rest is driven by physiological adaptations associated with migratory behaviour and habitat quality [60–62]. For instance, the amount of forest cover and productivity relates to a two-step mechanism determining the spatio-temporal use of stopover areas, via identification of high-quality habitat prior to landing and sustained refuelling rates during stopover [61,63]. Overall, stopover areas are important to replenish energetic reserves and can influence subsequent flight ranges [64,65]. However, stopover sites can have functions different from providing food, and their ecological context (e.g. proximity to ecological barriers, spatial isolation) and intrinsic characteristics (e.g. diversity and abundance of resources) may determine their use by migrants [23,34,62,66,67].

Most individuals do not use Ponza for refuelling [34], as they might just need to rest before continuing the last part of their journey [68,69]. Therefore, coexistence at stopover areas might not be regulated exclusively by avoidance of competition for resources, but also by the need to reduce the risk of predation or to gain information on novel habitats and resources [20,70,71]. Almost all species investigated in our study have a strictly carnivorous diet and feed mainly on insects but have nevertheless variable foraging niches. The only exceptions are the European turtle dove (*Streptopelia turtur*) and the Eurasian golden oriole (*O. oriolus*), which can be classified as granivore ground feeding and omnivorous arboreal, respectively [51]. Several studies have shown that species with similar diets switch to alternative foraging substrates and different predatory behaviour to avoid competition [51,72]. Our findings show no association between co-migration fidelity and foraging strategies, suggesting the existence of species-independent foraging mechanisms promoting coexistence at stopover areas.

However, if refuelling is not the main reason for migrants to land in an area [23], social (facilitating) interactions could explain the observed differences in species co-migration fidelity. Social interactions have been observed in migrating songbirds that use interspecific calls as cues to estimate stopover quality [20]. More recently, Gayk *et al*. [73] showed how warblers that migrate in mixed-species flocks produce flight calls to reduce disorientation during migration or to increase the chance of finding high quality stopover sites, a pattern even more pronounced in phylogenetically close species that breed at the same latitude with overlapping migration timing. Thus, it could be hypothesized that the observed tendency to migrate in well-defined and consistent subgroups of species with synchronized migration times is functional to optimize the costs associated with predation risks *en route*, resting at stopover areas, and ability to compete for territories at breeding areas.

## 5. Conclusion

Our study represents a first attempt in modelling the migratory timing of multiple bird species to gain new insights on the consequence of long-term changes in migration phenology on the restructuring of avian assemblages *en route*. Here, we introduce the concept of co-migration fidelity, a novel way to quantify the consistency and synchronization of migratory patterns using bird ringing data as building blocks in a methodological framework rooted in network theory. As animal migrations involve the simultaneous movements of individuals and species in space and time, long-term changes in migration timing have the potential to determine when, how long and with whom each species co-occurs with other members of the community. Our findings show that during spring migration African–European migratory landbirds follow a well-defined schedule, which we hypothesize to be likely driven by common responses to the effects of climate change on the timing of migration, a pattern likely to increase over time and mainly affecting species with low phenotypic plasticity covering longer distances during migration. The proposed approach can be applied to data of different origins, possibly extending the spatio-temporal coverage to species’ co-occurrence at wintering, stopover and breeding areas, to better understand the consistency of observed patterns in different migratory systems. This could help deepen the knowledge about how the effects of climate change on migratory phenology could influence species interactions, facilitating conservation decisions and serving as a basis for future research efforts in ecosystem ecology.

## Data Availability

Data and codes used in this paper are available at Figshare. Bird data: https://doi.org/10.6084/m9.figshare.23742060 [74]; R code: https://doi.org/10.6084/m9.figshare.23742132 [75].
